# Supplementary material for: Combinatorial immunotherapy of anti-MCAM CAR-modified expanded natural killer cells and NKTR-255 against neuroblastoma
Source: Mol Ther Oncol. 2024 Oct 18;32(4):200894. doi: 10.1016/j.omton.2024.200894 (PMC11567912; doi:10.1016/j.omton.2024.200894)
Supplement: Document S1. Figures S1–S4 [file mmc1.pdf]

OMTON, Volume *32*

Supplemental information

Combinatorial immunotherapy of anti-MCAM CAR-modified expanded natural killer cells and NKTR-255 against neuroblastoma

Wen Luo, Aliza Gardenswartz, Hai Hoang, Yaya Chu, Meijuan Tian, Yanling Liao, Janet Ayello, Jeremy M. Rosenblum, Xiaokui Mo, A. Mario Marcondes, Willem W. Overwijk, Timothy P. Cripe, Dean A. Lee, and Mitchell S. Cairo

Supplemental Information

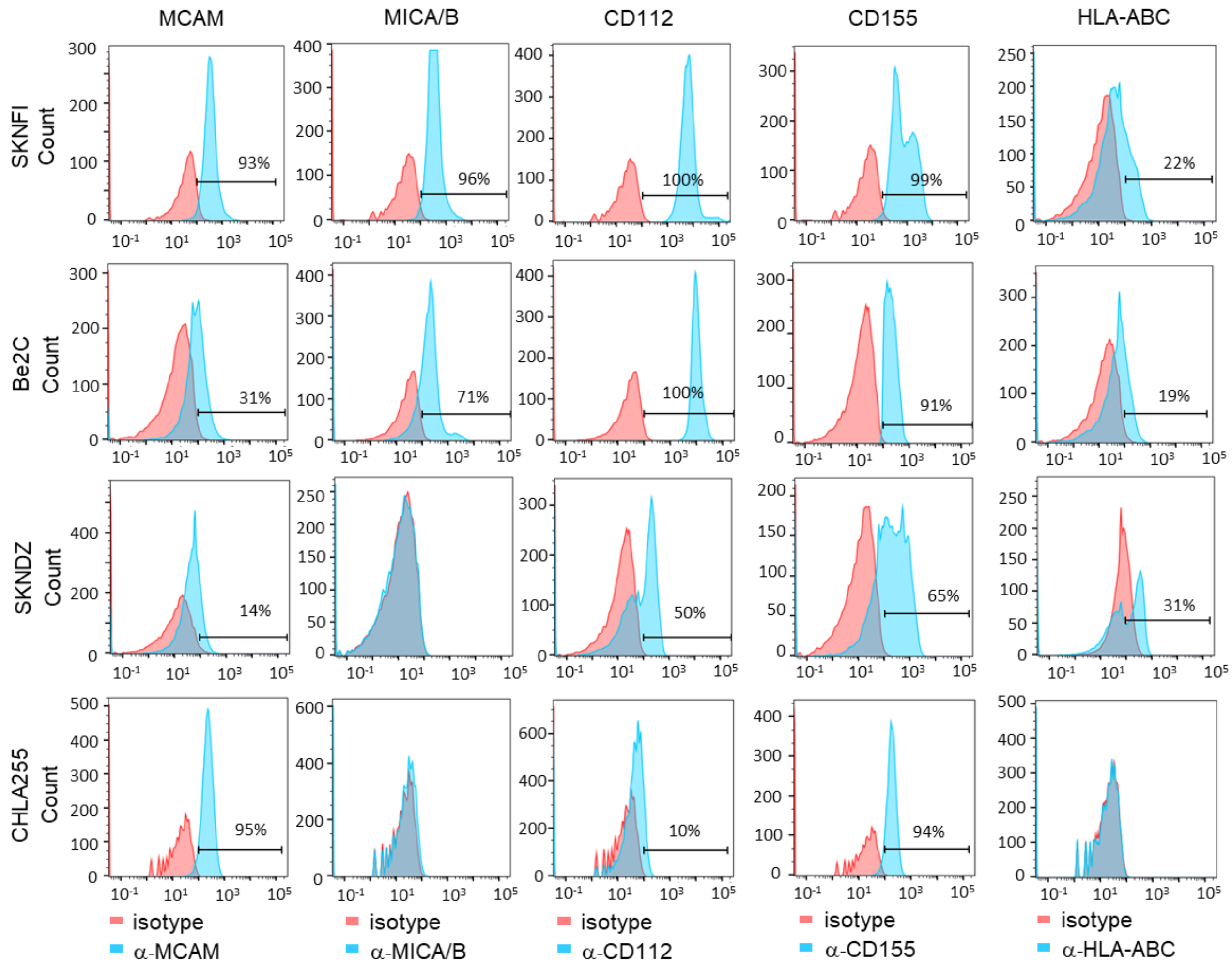

Figure S1. Representative flow histograms of MCAM and NK receptor ligands showing expression on the surface of NB cell lines. SK-N-FI, Be2C, SK-N-DZ and CHLA255 cells were stained with isotypes or flow antibodies against MCAM or NK activating receptor ligands MICA/B, CD112, CD155, or NK inhibitory receptor ligands HLA-ABC, and subject to flow cytometry analyses.

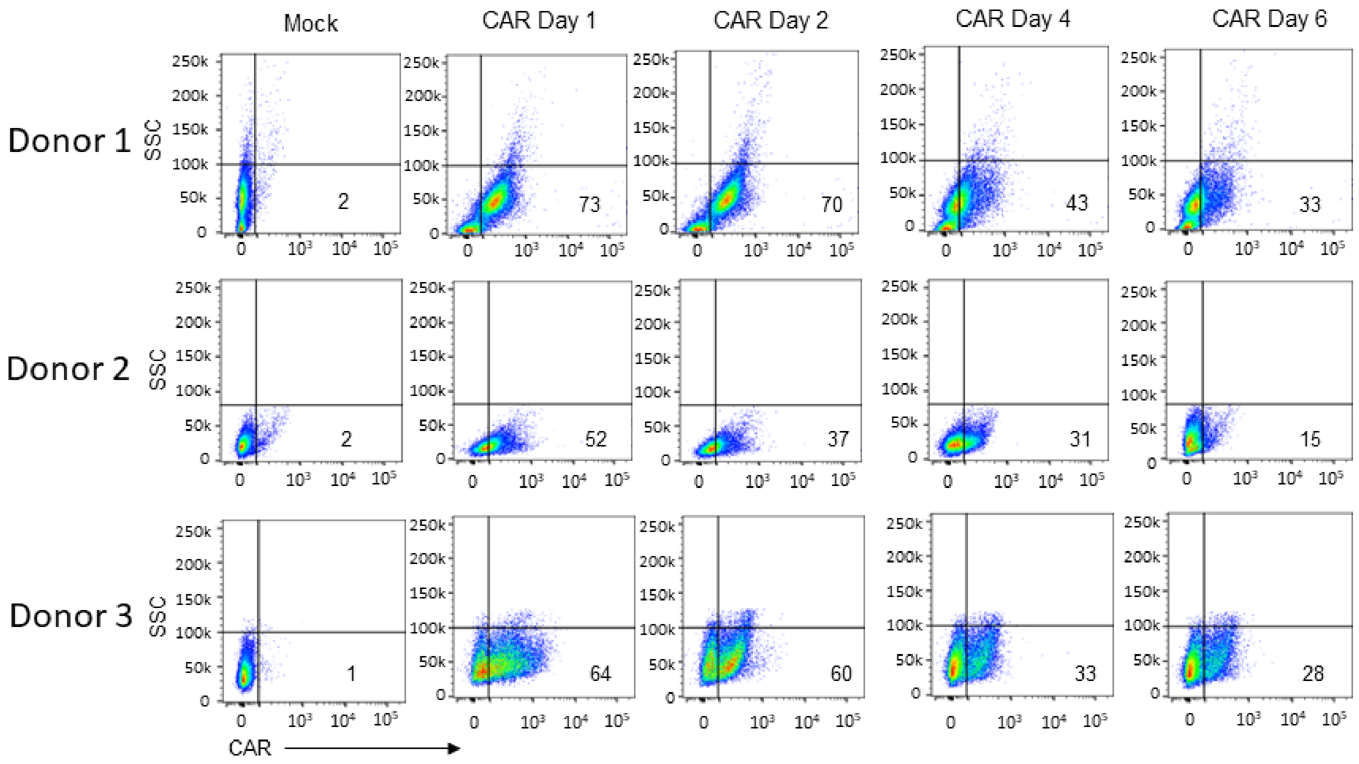

Figure S2. Electroporation mediated CAR expression on ex-vivo expanded NK cells from 3 different donors. Ten micrograms of in vitro transcribed CAR mRNA were introduced into $5x10^6$ exNK cells using electroporation. Twenty four to 48 hours after electroporation, CAR expression was detected by biotinylated MCAM protein followed by FITC-streptavidin staining and flow cytometry. SSC-FITC dot plots are shown. The numbers are percent of CAR positive population in the total cells.

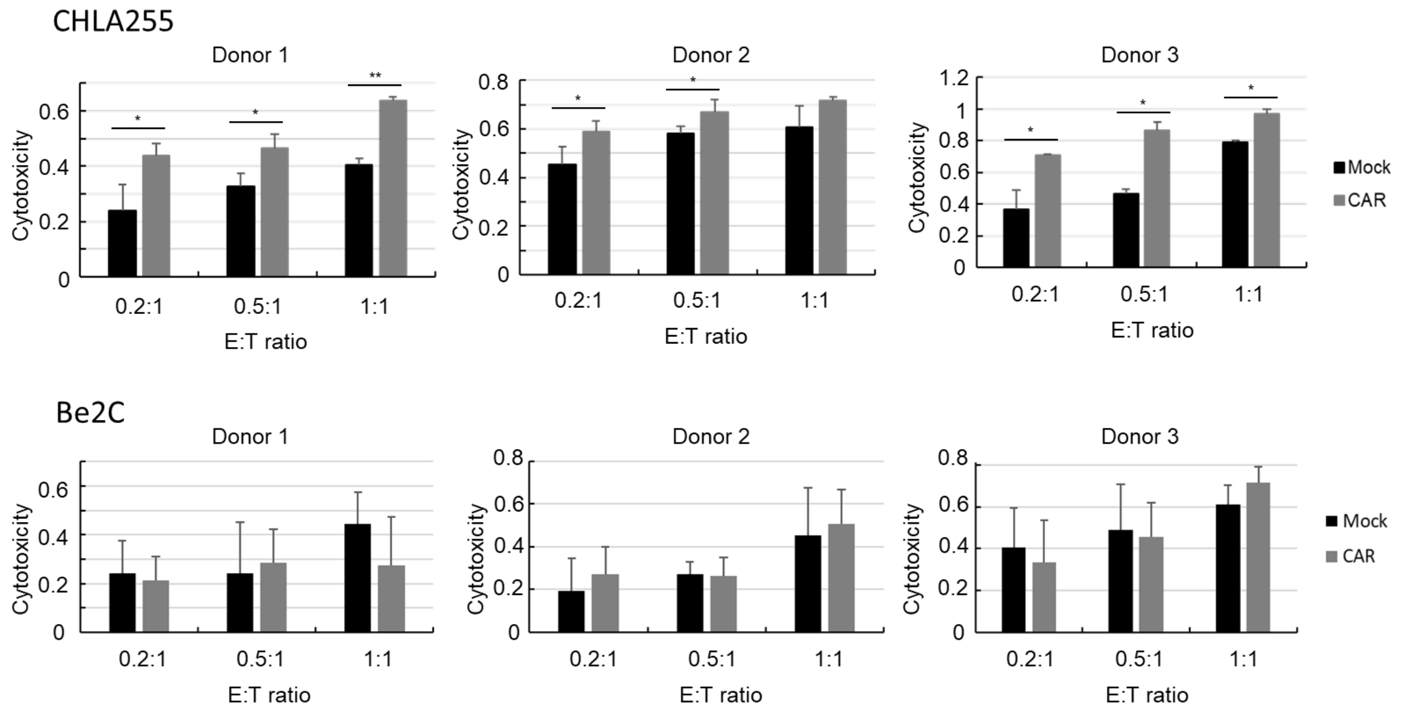

Figure S3. MCAM CAR NK cell cytotoxic activity against NB CHLA255 and Be2C cells compared to the mock NK cells. Columns represent the mean values; error bars indicate the standard deviation of triplicate samples in a representative experiment. *p < 0.05, **p < 0.01. The same trend was seen using three different donors derived NK cells.

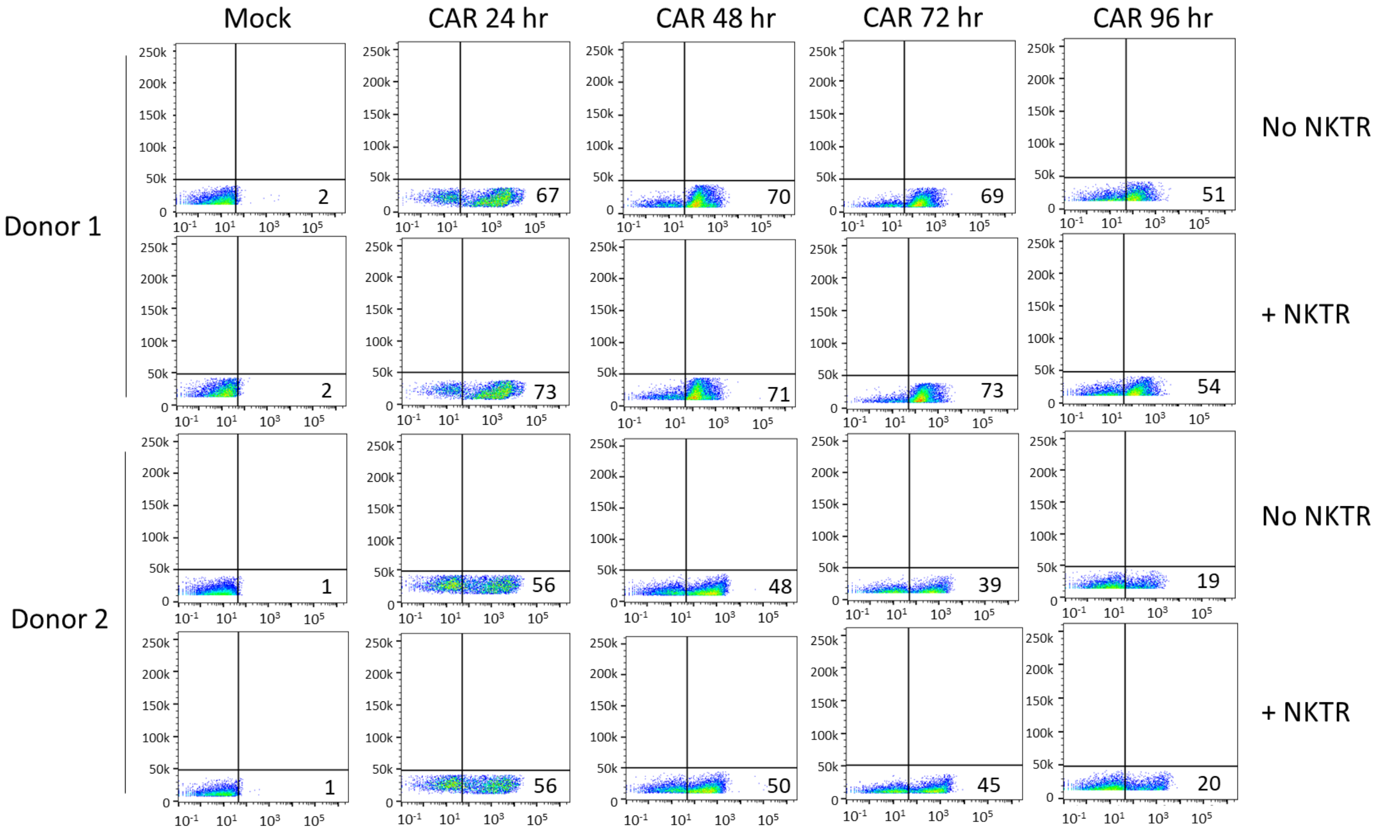

Figure S4. Effects of NKTR-255 on MCAM CAR expression level and duration on ex-vivo expanded NK cells. NK cells were electroporated with CAR mRNA and incubated with NKTR-255 (40 ng/mL) in the absence of IL-2 in RPMI media for 96 hours. CAR NK cells were sampled every 24 hours and CAR expression was detected by biotinylated MCAM protein followed by FITC-streptavidin staining and flow cytometry. SSC-FITC dot plots are shown. The numbers are the percent of CAR positive population in the total cells.

Supplemental Material

Cell lines

NB cell lines SK-N-FI, Be2C and SK-N-DZ were obtained from American Type Culture Collection (ATCC) and grown according to ATCC recommendations. CHLA-255 cells were

generously provided by Robert Seeger, MD from Children's Hospital Los Angeles, CA, USA. Cell lines are tested quarterly for mycoplasma contamination using MycoProbe Mycoplasma Detection Kit (CUL001B, R&D Systems, Minneapolis, MN, USA) and cells are authenticated by short tandem repeat profiling (Genetica DNA Laboratories, Cincinnati, OH, USA).

DNA constructs

The MCAM Ab single chain variable fragment (scFv) sequence was generously provided by Bin Liu (University of California at San Francisco, United States)[29] and the scFv DNA was codon optimized and synthesized (Integrated DNA Technologies, Coralville, IA, USA) followed by subcloning in-frame with a linker (eskygppcppcpm), CD28TM (NP_001230007.1, aa 34-59), 4-1BB (NP_001552, aa 213-255), and CD3ζ (NP_000725, aa 52-163) into the pcDNA3 vector to generate a second generation anti-MCAM CAR. A 2bgUTR.150A sequence generously provided by Carl June and Yangbing Zhao (University of Pennsylvania)[30] was inserted at 3' end of the CAR construct to further optimize the construct. The clustered regulatory interspaced short palindromic repeats (CRISPR)/Cas9 knockout constructs for MCAM were created by cloning the CRISPR guide RNA against MCAM (5'-GTTGCATGACCTGAAACGGG-3' and 5'-AGGAGGCGGCTATCGCTGCG-3') into the plentiCRISPRv2 vector (Addgene plasmid #52961).[31] Guide sequences were designed using the Broad Institute sgRNA designer tool (https://portals.broadinstitute.org/gpp/public/analysis-tools/sgrna-design).

Ex-vivo NK expansion

NK cells were expanded using donor peripheral blood mononuclear cells and irradiated K562-mbIL21-41BBL feeder cells and purified by using a NK cell isolation kit (Miltenyi Biotec, 130-092-657, Bergisch Gladbach, Germany) as we have previously described.[8]

CAR mRNA electroporation

Anti-MCAM CAR mRNA was in-vitro transcribed using the mMESSAGE mMACHINE T7 ultra transcription kit (Thermo Fisher Scientific, AM1345, Waltham, MA, USA) according to the manufacturer's instructions. Expanded NK cells ($5x10^6$) were electroporated with anti-MCAM CAR mRNA (10 µg) or H_2O (negative control) using the MaxCyte GT® electroporation system (MaxCyte Inc. Rockville, MD, USA). Anti-MCAM CAR mRNA electroporation efficiency was evaluated by flow cytometry using biotinylated MCAM protein (HY-P75613, MedChemExpress, Monmouth Junction, NJ, USA) followed by FITC-streptavidin (405202, BioLegend, San Diego, CA, USA). Anti-MCAM CAR expression was detected at day 1, 2, 4, and 6 post electroporation.

Bioluminescence (BLI) based in-vitro cytotoxicity assay

BLI based in-vitro cytotoxicity assays were performed as we have previously described[27] with minor modifications. Luciferase-expressing tumor cells ($5x10^4$) were incubated with effector cells (NK/CAR NK) at different effector-to-target (E:T) ratios (0.2:1, 0.5:1, and 1:1) in DMEM media supplemented with 10% FBS in 96–well tissue culture plates at 37°C for 4 hours before D-firefly luciferin potassium salt (LUCK-1G, Goldbio, St Louis, MO, USA) was added to the cells and BLI was measured with a luminometer (Molecular Devices Multifilter F5 plate reader). In CAR NK and NKTR-255 combination cytotoxicity assay, MCAM CAR NK cells were cultured in RPMI1640 media supplemented with or without NKTR-255 (40 ng/ml, Nektar

Therapeutics, San Francisco, CA, USA) for 72 hours before incubating with luciferase expressing tumor cells.

Flow cytometry

Cells ($1x10^6$ single cell suspension) were washed once with ice cold FACS buffer (DPBS, 0.5% BSA) and blocked with 100 μL of Fc block (BD Biosciences, 564219, Franklin Lakes, NJ, USA) diluted in FACS buffer at 1:50 ratio at 4°C for 20 min. Cells were then collected by centrifugation at 1500 rpm for 5 min and resuspended in 100 μL fluorescent conjugated primary antibody in FACS buffer (for antibody concentration see Supplementary Table 1) and incubated at 4°C for 1 hour in the dark. Cells were washed twice and resuspended in 100 μL ice cold FACS buffer. If cells were stained with unlabeled primary antibody, cells were resuspended in fluorescent conjugated secondary antibody in FACS buffer at the dilution ratio recommended by the manufacturer (see Supplementary Table 1 for details) and incubated at 4°C in the dark for 30 min. Cells were then washed twice and resuspended in 100 μL ice cold FACS buffer. Flow cytometry analyses were performed using FACSCelesta Cell Analyzer and data were analyzed by FACSDeva (BD Biosciences, Franklin Lakes, NJ, USA) and Flowjo (FlowJo LLC, Ashland, OR, USA).

Enzyme-linked immunosorbent assay (ELISA)

IFN-γ (430104, BioLegend, San Diego, CA, USA) and Perforin (ab46068, Abcam, Cambridge, UK) concentrations were analyzed by ELISA according to the manufacturer's instructions as we have previously described.[27]

In-vitro treatment of NK cells with NKTR-255

ExNK cells were isolated from day 14 of expansion culture (using IL-2 and irradiated feeder cells) from three donors by NK cell isolation kit (Miltenyi Biotec, 130-092-657, Bergisch Gladbach, Germany). Isolated NK cells ($2.5x10^6$ per condition) were then incubated with 0, 1, 10, or 40 ng/mL of NKTR-255 in RPMI 1640 media for 6 days. The number of viable cells were counted every 24 hours using trypan blue staining method.

Statistical analyses

Analysis of variance (ANOVA) was used to analyze experiments with multiple independent groups. In-vivo tumor growth was analyzed by mixed effect model, accounting for observational dependencies for each subject. ANOVA and mixed effect modeling were conducted using SAS 9.4 (SAS Institute, Cary, NC, USA). Before conducting mouse experiments, sample sizes achieving 80% power to detect an effect size >2 were determined at significant level as 0.05 using PASS 20 (Power Analysis and Sample Size Software. NCSS, LLC.). All data are presented as the mean ± SD of at least three independent experiments except where stated.
