# Supplementary material for: Combinatorial immunotherapy of anti-MCAM CAR-modified expanded natural killer cells and NKTR-255 against neuroblastoma
Source: Mol Ther Oncol. 2024 Oct 18;32(4):200894. doi: 10.1016/j.omton.2024.200894 (PMC11567912; doi:10.1016/j.omton.2024.200894)
Supplement: Document S2. Article plus supplemental information [file mmc2.pdf]

Molecular Therapy
Oncology
Brief report

Combinatorial immunotherapy of anti-MCAM CAR-modified expanded natural killer cells and NKTR-255 against neuroblastoma

Wen Luo,[1,2] Aliza Gardenswartz,[1] Hai Hoang,[1] Yaya Chu,[1] Meijuan Tian,[1] Yanling Liao,[1] Janet Ayello,[1] Jeremy M. Rosenblum,[1] Xiaokui Mo,[3] A. Mario Marcondes,[4] Willem W. Overwijk,[4] Timothy P. Cripe,[5,6] Dean A. Lee,[5,6,9] and Mitchell S. Cairo[1,2,7,8,9]

[1]Department of Pediatrics, New York Medical College, Valhalla, NY 10595, USA; [2]Department of Pathology, Immunology and Microbiology, New York Medical College, Valhalla, NY, USA; [3]Center for Biostatistics, Department of Biomedical Informatics, The Ohio State University, Columbus, OH 43210, USA; [4]Nektar Therapeutics, San Francisco, CA 94158, USA; [5]Department of Pediatrics, College of Medicine, The Ohio State University, Columbus, OH, USA; [6]Center for Childhood Cancer Research, Abigail Wexner Research Institute at Nationwide Children's Hospital, Columbus, OH 43205, USA; [7]Department of Medicine, New York Medical College, Valhalla, NY, USA; [8]Department of Cell Biology and Anatomy, New York Medical College, Valhalla, NY, USA

Pediatric patients with recurrent metastatic neuroblastoma (NB) have a dismal 5-year survival. Novel therapeutic approaches are urgently needed. The melanoma cell adhesion molecule (MCAM/CD146/MUC18) is expressed in a variety of pediatric solid tumors, including NB, and constitutes a novel target for immunotherapy. Here, we developed a chimeric antigen receptor (CAR) expressing natural killer (NK) cell-targeting MCAM by non-viral electroporation of CAR mRNA into *ex vivo* expanded NK cells. Expression of anti-MCAM CAR significantly enhanced NK cell cytotoxic activity compared to mock NK cells against MCAM^{high} but not MCAM$^{low/knockout}$ NB cells *in vitro*. Anti-MCAM-CAR-NK cell treatment significantly decreased tumor growth and prolonged animal survival in an NB xenograft mouse model. NKTR-255, a polymer-conjugated recombinant human interleukin-15 agonist, significantly stimulated NK cell proliferation and expansion and further enhanced the *in vitro* cytotoxic activity and *in vivo* anti-tumor efficacy of anti-MCAM-CAR-NK cells against NB. Our preclinical studies demonstrate that *ex vivo* expanded and modified anti-MCAM-CAR-NK cells alone and/or in combination with NKTR-255 are promising novel alternative therapeutic approaches to targeting MCAM^{high} malignant NB.

INTRODUCTION

Neuroblastoma (NB) is the most common malignant solid tumor that occurs in infants.[1] Patients with recurrent metastatic NB have a dismal survival.[2] Current treatments, including surgery, radiation, high-dose chemotherapy, and autologous stem cell transplantation, have failed to improve patient outcome in advanced disease.[3–6] Novel therapies are urgently needed.

Natural killer (NK) cells have long been recognized as important in treating pediatric solid tumors, including NB.[7] Unlike T cells, NK cells target tumor cells without requiring prior sensitization or specific antigen recognition and have not been associated with significant cytokine-related side effects and neurotoxicity. To enhance the cytotoxicity of NK cells and facilitate specific targeting of tumor cells, we and others have engineered NK cells to express chimeric antigen receptors (CARs) against molecular targets.[8,9] However, due to the scarcity of tumor-associated antigens in pediatric solid tumors,[10] only few NK cell CARs have been developed to target NB.[11]

Melanoma cell adhesion molecule (MCAM) is a cell-surface protein overexpressed in a variety of pediatric cancers, including NB.[10] Increased MCAM expression is associated with poor prognosis, increased metastasis, and recurrence rate in various cancers.[12] MCAM is highly expressed in the embryo, but its expression in mature normal tissue is minimal.[13] These features make MCAM a promising target for immunotherapy for patients with metastatic MCAM+ solid tumors. A fully humanized anti-MCAM antibody, ABX-MA1, was found to inhibit spontaneous pulmonary metastasis of osteosarcoma in an orthotopic mouse model.[14] However, MCAM-targeted cellular immunotherapy against NB has not been investigated previously.

Two limitations of NK cell tumor immunity is the low number and poor persistence of NK cells *in vivo*. NKTR-255 is an investigational polymer-conjugated, recombinant human interleukin-15 (IL-15) receptor agonist that activates the IL-15 pathway and stimulates

Received 1 May 2024; accepted 15 October 2024;
https://doi.org/10.1016/j.omton.2024.200894.

[9]Senior author

Correspondence: Dean A. Lee, Department of Pediatrics, College of Medicine, The Ohio State University, Columbus, OH, USA.
E-mail: dean.lee@nationwidechildrens.org
Correspondence: Mitchell S. Cairo, Department of Pediatrics, New York Medical College, Valhalla, NY 10595, USA.
E-mail: mitchell_cairo@nymc.edu

© 2024 The Author(s).
Published by Elsevier Inc. on behalf of The American Society of Gene and Cell Therapy.
This is an open access article under the CC BY license (http://creativecommons.org/licenses/by/4.0/).

proliferation and survival of NK and CD8+ T cells[15] and is currently in early-phase clinical trials with monoclonal antibodies or CAR T cell therapy (ClinicalTrials.gov: NCT04136756, NCT03233854, and NCT04616196), as reported by our group and others.[16]

In the current study, we engineered an *ex vivo* expanded CAR NK cell targeting MCAM and combined it with NKTR-255 in order to circumvent some of the limitations of NK cell therapy to facilitate increased anti-tumor efficacy against NB.

RESULTS

To evaluate the potential of NB cells to engage the NK cell activation or inhibitory receptors, we investigated the expression levels of the respective ligands, MHC class I chain-related protein A and B (MIC A/B), CD112, CD155, and human leukocyte antigen-ABC (HLA-ABC), in the NB cell lines SK-N-FI, Be2C, SK-N-DZ, and CHLA-255. We found that SK-N-FI and Be2C cells express intermediate levels of MIC A/B, high levels of CD112 and CD155, and low levels of HLA-ABC; CHLA-255 cells express high levels of CD155 and low levels of HLA-ABC (Figure S1). This suggests that these cells have the potential to induce more activating signals than inhibitory signals in NK cells, which renders them susceptible to NK cell cytotoxicity. SK-N-DZ cells, on the other hand, express a very low level of MIC A/B and an intermediate level of HLA-ABC, suggesting low sensitivity of these cells to NK cells. To evaluate MCAM as a target for CAR against NB cells, we analyzed MCAM expression levels in these cells and detected high levels of MCAM expression on SK-N-FI and CHLA-255 cells. In contrast, Be2C and SK-N-DZ cells express an intermediate and a low level of MCAM, respectively (Figure S1).

To assess cytotoxic activity of anti-MCAM-CAR-NK cells against NB cells, we engineered K562-membrane bound IL-21 (mbIL-21)-41BBL expanded NK (exNK) cells to express CARs against MCAM (Figures 1A and 1B). Electroporation of the CAR mRNA into exNK cells resulted in CAR expression in >60% of NK cells (Figure 1B), and the CAR expression lasted for at least 6 days (Figures 1C and S2). We performed *in vitro* cytotoxicity assays 1–2 days post CAR mRNA electroporation, when CAR expression on exNK cells was at the peak, and used SK-N-FI, CHLA-255, and Be2C cells, which express high and intermediate levels of MCAM and are potentially sensitive to NK cell cytotoxicity, as target cells. We found that, compared to the unmodified exNK cells (mock), expression of anti-MCAM CARs in exNK cells (CAR) significantly enhanced the NK cell cytotoxicity against the MCAM^{high} SK-N-FI and CHLA-255 cells but not the Be2C cells (Figures 1D and S3) at effector-to-target (E:T) ratios of 0.2:1 and 0.5:1. We observed the significant differences between mock and CAR NK cells mostly at low E:T ratios (0.2:1 and 0.5:1), likely due to the high sensitivity of these tumor cells to NK cells at baseline. Next, we compared mock and CAR NK cell responses to SK-N-FI cells and found that CAR NK cells had significantly higher cytokine (interferon γ and perforin) secretion than mock NK cells when incubated with SK-N-FI cells (Figure 1E). To investigate whether the enhanced cytotoxic activity of CAR NK cells was due to specific targeting of MCAM, we knocked out MCAM in SK-N-FI cells (Figure 1F) and compared the cytotoxic activity of mock and CAR NK cells against wild-type (WT) and MCAM knockout (KO) cells. In the KO tumor cells, we did not observe a significant increase in cytotoxicity with CAR NK cells compared to mock NK cells as we did in the WT tumor cells (Figure 1G).

Next, we investigated the efficacy of anti-MCAM-CAR-NK cells in limiting NB xenograft tumor growth *in vivo* in NB (SK-N-FI)-xenografted NSG (NOD.Cg-*Prkdc*^{scid} *Il2rg*^{tm1Wjl}/SzJ) mice. Consistent with the *in vitro* data, we found that treatment with CAR-modified exNK cells significantly decreased NB xenograft tumor growth compared to the vehicle and mock NK cells (Figure 1H) and prolonged animal survival compared to the vehicle (Figure 1I). Compared to the mock NK cell-treated mice, which all died at the end of the study (day 95), 30% of the mice treated with CAR NK cells were still alive (Figure 1I).

To improve CAR NK cell survival and persistence, we employed an IL-15 agonist, NKTR-255. We found that NKTR-255 treatment (40 ng/mL, 96 h) markedly increased the expression levels of NK cell-activating receptors including NKp30, natural killer group 2 member D (NKG2D), and NKp44, in the absence of IL-2 compared to the untreated exNK cells and only induced a mild increase in NKG2A and killer Ig-like receptors (KIR) expression levels (Figure 2A). Furthermore, NKTR-255 significantly improved NK cell survival and maintained NK cell expansion in the absence of IL-2 *in vitro* (Figure 2B). We therefore investigated the effect of NKTR-255 on the anti-tumor activity of the MCAM-CAR-NK cells. We first investigated whether NKTR-255 had any effect on CAR expression and observed no significant changes in CAR expression level or duration after 24, 48, 72, or 96 h of NKTR-255 treatment (Figure S4). We found that NKTR-255 significantly enhanced the cytotoxic activity of MCAM-CAR-NK cells targeting SK-N-FI cells at an E:T ratio of 0.5:1 (Figure 3A) and significantly enhanced secretion of interferon γ (Figure 3B) and perforin (Figure 3C) from MCAM-CAR-NK cells when incubated with SK-N-FI cells. In the NB (SK-N-FI)-xenografted mice, NKTR-255 alone did not have a significant effect on NB xenograft tumor growth or animal survival. However, combination with NKTR-255 further enhanced the anti-tumor effects of MCAM-CAR-NK cells ($p < 0.001$ compared to vehicle control, $p < 0.05$ compared to CAR NK cells) and further prolonged animal survival (100% vs. 30% survival on day 126 compared to CAR NK cells, $p < 0.05$) (Figures 3D–3F).

DISCUSSION

While CAR T cells and checkpoint inhibitors have been successful in other pediatric and adult malignancies, these approaches in NB are severely limited,[17] in part due to low expression of major histocompatibility complex (MHC) molecules, low mutational burden in NB, and severe side effects, including cytokine release syndrome and immune effector cell-associated neurotoxicity syndrome.[18] An alternative approach is to utilize NK cells that demonstrate anti-tumor cytolytic activity in an MHC-independent manner. *In vitro* data from us and others have indicated that NB cell lines are highly susceptible to

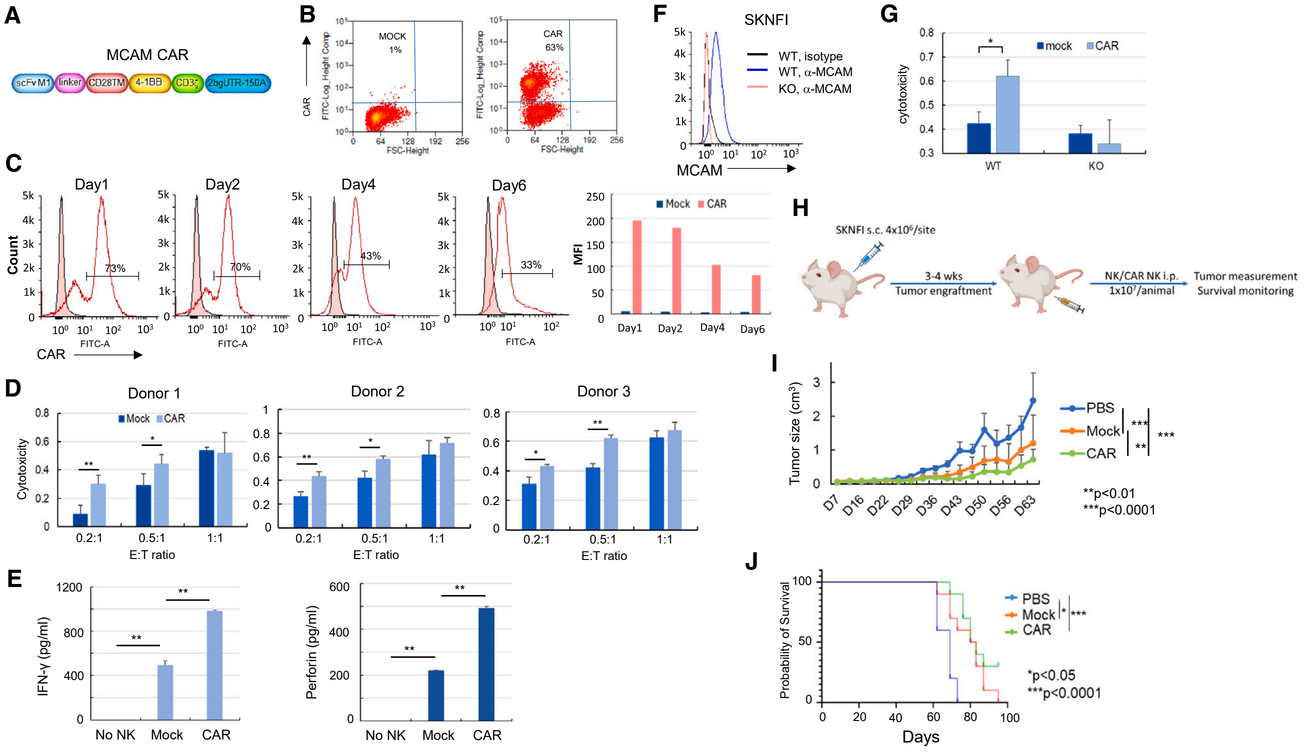

Figure 1. Anti-MCAM-CAR-NK cells had superior cytotoxic activity against MCAM^{high} NB cells *in vitro* and enhanced anti-tumor activity in NB xenograft mouse model compared to mock NK cells

(A) Schematic of the design of the anti-MCAM CAR construct. (B) CAR expression on *ex vivo* exNK cells mediated by electroporation of CAR mRNA. Ten micrograms of *in vitro*-transcribed CAR mRNA was introduced into 5 × 10^{6} exNK cells using electroporation. Forty-eight hours after electroporation, CAR expression was detected by biotinylated MCAM protein, followed by fluorescein isothiocyanate (FITC)-streptavidin staining and flow cytometry. (C) Duration of expression of CAR on *ex vivo* exNK cells. CAR expression was detected 1, 2, 4, and 6 days post electroporation. Median fluorescence intensity for the CAR+ population was analyzed by FlowJo. (D) Compared to the unmodified exNK cells (mock), expression of anti-MCAM CAR in exNK cells (CAR) significantly enhanced the NK cytotoxic activity against MCAM^{high} NB SK-N-FI cells. $^{*}p < 0.05$, $^{**}p < 0.01$ (two-tailed Student's t test). Columns represent the mean values, and error bars indicate the standard deviation (SD) of triplicate samples in a representative experiment. The same trend was seen using three different donor-derived NK cells. (E) Compared to the mock NK cells, the anti-MCAM-CAR-NK cells (CAR) had significantly increased secretion of the cytokines interferon γ (IFN-γ; left) and perforin (right). $^{*}p < 0.05$, $^{**}p < 0.01$ (two-tailed Student's t test). Columns represent the mean values, and error bars indicate the SD of triplicate samples in a representative experiment. The same trend was seen in three independent biological replicates. (F) Flow cytometry analysis showing MCAM KO by the CRISPR-Cas9 approach in SK-N-FI cells. (G) Specific targeting of MCAM^{high} SK-N-FI cells by anti-MCAM-CAR-NK cells. Cytotoxic activities of mock and CAR NK cells against MCAM wild-type (WT) and knockout (KO) cells were compared. Columns indicate the mean, and error bars indicate the SD of triplicate samples in a representative experiment. $^{*}p < 0.05$, $^{**}p < 0.01$ (two-tailed Student's t test). The same trend was seen in three independent biological replicates. (H) Schematic showing the *in vivo* study design and procedure in (I) and (J). NB SK-N-FI cells (4 × 10^{6} cells/site) were implanted subcutaneously into 4- to 6-week-old female NSG mice. After tumor establishment, 1 × 10^{7} of NK or CAR NK cells in PBS were injected intraperitoneally once a week for 5 weeks. Tumor growth was monitored by caliper measurement, and animals were followed until death or sacrificed upon reaching a tumor size of 2 cm in any dimension. (I) Anti-MCAM-CAR-NK treatment significantly decreased tumor growth in an NB xenograft mouse model. Mock NK or anti-MCAM-CAR-NK cells or PBS control were injected into NB xenograft tumor-bearing NSG mice intraperitoneally once a week for 5 weeks. Tumor growth was monitored by caliper measurement twice a week. Tumor size was estimated according to the following formula: tumor size (cm^{3}) = length (cm) × width^{2} (cm) × 0.5. $n = 10$ per group. $^{**}p < 0.01$, $^{***}p < 0.0001$ (ANOVA). Growth rates between groups were analyzed using a mixed-effects model. (J) Anti-MCAM-CAR-NK cell treatment significantly prolonged animal survival in the NB xenograft mouse model. Mice were followed until death or sacrificed if any tumor size reached 2 cm in any dimension in (I). Probability of survival was determined by the Kaplan-Meier method using animal death/sacrifice as the terminal event using Prism v.8.0 (GraphPad). $^{*}p < 0.05$, $^{***}p < 0.0001$ (log rank test).

NK cell-mediated cell lysis due to the low expression of MHC and high expression of NK cell-activating receptor ligands (Figure S1).[19] Consistent with this, increased NK cell frequency in NB patient tumors has been associated with a favorable prognosis.[20]

However, NK cell number and function are low in patients with NB tumors, largely due to the low number of active NK cells; poor NK cell function, activation, and persistence; and lack of specific targeting, among others.[21] To address low NK cell number and function, our group has developed a genetically engineered antigen-presenting cell (K562) expressing mbIL-21 and 4-1BBL to expand peripheral blood mononuclear cells into NK cells.[22] This approach results in over 35,000-fold expansion in NK cells and significant NK cell functional activation.[22]

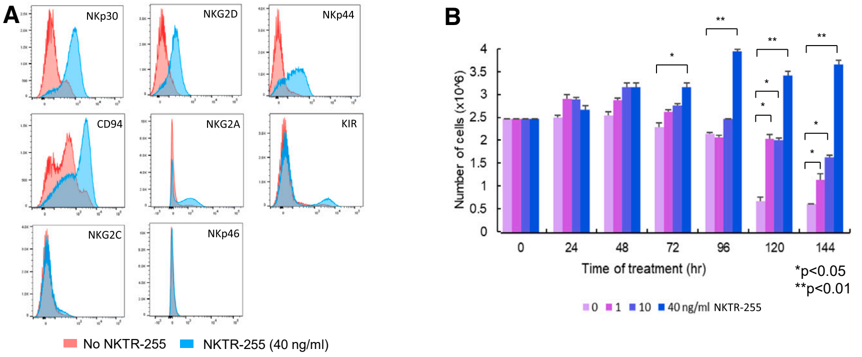

Figure 2. NKTR-255 treatment increased the expression of NK-activating receptors and stimulated NK cell proliferation and expansion *in vitro*
(A) Expression of NKp30, NKG2D, NKp44, CD94, NKG2A, KIR, NKG2C, and NKp46, detected by flow cytometry on *ex vivo* exNK cells treated with or without NKTR-255 (40 ng/mL) for 96 h in the absence of IL-2. (B) *Ex vivo* exNK cells (2.5 × 10^6 cells per condition) were incubated with increasing concentrations of NKTR-255 (0, 1, 10, and 40 ng/mL) in the absence of IL-2 for various periods of time as indicated (0–144 h). The viable cells were counted every 24 h using the trypan blue staining method. *$p < 0.05$, **$p < 0.01$. Columns indicate mean values, and error bars indicate the SD of triplicate samples in a representative experiment. The same trend was seen in three independent biological repeats.

To enhance NK cell targeting specificity, we developed an mRNA electroporation technology to efficiently engineer exNK cells to express CARs.[8] We and others have demonstrated that CAR NK or T cells generated by the mRNA electroporation approach are effective and safe without neurologic or systemic toxicity in lymphoma, medulloblastoma, and high-grade glioma.[8,23,24] Here, we demonstrated significantly enhanced *in vitro* cytotoxic activity and *in vivo* anti-tumor efficacy of MCAM-targeting CAR NK cells against NB compared to mock NK cells (Figure 1). NB is an extremely heterogeneous tumor.[25] We observed heterogeneity in MCAM and NK cell receptor ligands expression in our study (Figure S1). SK-N-FI and CHLA-255 cells, which express high levels of MCAM and NK cell-activating receptor ligands, were sensitive to the MCAM-CAR-NK cells, while Be2C and SK-N-DZ cells were not, due to their relatively low expression of MCAM or high expression of NK cell-inhibitory receptor ligands. This is consistent with a previous report that low surface density of the target (CD22), rather than total loss of its expression on tumor cells, is sufficient to allow escape from CAR therapy.[26] This underscores the importance of monitoring the target expression level on patient tumors before and during the CAR therapy. In addition, these results, together with the cytotoxicity data for MCAM KO cells (Figure 1G), demonstrated the targeting specificity of the MCAM-CAR-NK cells.

IL-15 plays important roles in stimulating the proliferation and cytolytic activity of NK cells and CD8+ T cells with limited effect on regulatory T cells.[21,27] Due to obstacles in production, a poor pharmacokinetic profile, weak potency, and its toxicity profile, the clinical application of recombinant human IL-15 (rhIL-15) has been limited.[21] IL-15 agonists have been developed to address these limitations. We have reported previously that N-803, consisting of an IL-15 agonist mutein (IL-15N72D) and a dimeric IL-15 receptor alpha (IL-15Rα)/Fc fusion protein, significantly increased the viability, proliferation, and antibody-dependent cellular cytotoxicity (ADCC) of exNK cells.[28] The combination of dinutuximab and N-803 significantly enhanced the *in vitro* cytotoxicity and *in vivo* anti-tumor effect of exNK cells against osteosarcoma, NB, and glioblastoma, as we have reported previously.[28] Different from N-803, NKTR-255 is a polyethylene glycol conjugate of rhIL-15 that exhibits a longer half-life, reduced clearance, and prolonged receptor affinity in comparison to rhIL-15.[15] Functionally, NKTR-255 induces the proliferation and activation of NK and CD8+ T cells, increases the CD8+:regulatory T cell ratio, increases the accumulation and persistence of anti-CD19 CAR T cells in the bone marrow, and synergizes with monoclonal antibodies to enhance ADCC in cancer models.[15,16] Consistent with our previous report, we found that NKTR-255 stimulated NK cell activation and maintained NK cell *ex vivo* expansion in the absence of IL-2 (Figure 2). By combining the specific and effective targeting of MCAM^{high} NB cells via anti-MCAM-CAR and the enhancement of NK *in vivo* persistence by NKTR-255, we were able to improve the therapeutic efficacy of NK cells against NB (Figure 3). Interestingly, the combinatorial effect of NKTR-255 and CAR NK cells was not due to increased CAR expression or duration because we did not observe significant effects of NKTR-255 on either (Figure S4).

Due to heterogeneous expression of MCAM on NB cell lines, we only evaluated the efficacy of MCAM-CAR-NK cells by utilizing a xenograft mouse model based on one NB cell line that expresses a high level of MCAM. In addition, the NSG mouse lacks mature murine T, B, and functional NK cells and is deficient in cytokine signaling. These characteristics are supportive of stable engraftment of human tumor cells and the evaluation of human NK cell anti-tumor efficacy. However, a validation of the study result in a syngeneic or humanized mouse model is warranted.

Nevertheless, our findings demonstrate that *ex vivo* expanded MCAM-CAR-NK cells are effective alone and with NKTR-255 in NB. MCAM-CAR-NK cells in combination with NKTR-255 are a promising novel alternative therapeutic approach for NB and potentially other MCAM^{high}-expressing malignancies.

MATERIALS AND METHODS

Additional methods are detailed in the supplemental information.[8,29–31]

Animal studies

All animal studies were performed in accordance with protocols approved by the New York Medical College Institutional Animal Care and Use Committee. SK-N-FI cells (4 × 10^6 cells/site) with

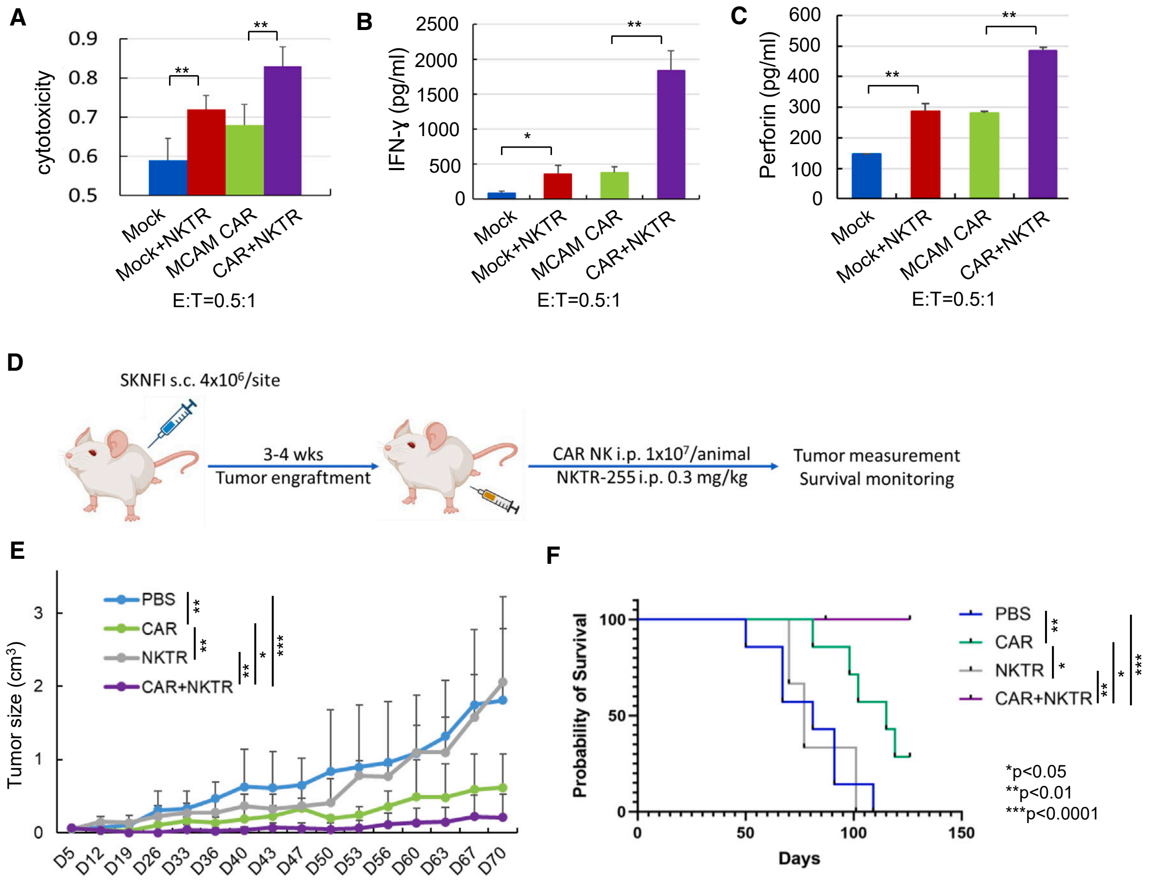

Figure 3. Combination of NKTR-255 significantly increased the cytotoxic activity of anti-MCAM-CAR-NK cells against NB cells *in vitro* and further enhanced the anti-tumor activity of anti-MCAM-CAR-NK cells *in vivo* in the NB xenograft mouse model

(A–C) Mock NK or MCAM-CAR-NK cells were incubated with or without 40 ng/mL NKTR-255 for 72 h, followed by *in vitro* cytotoxicity assays against SK-N-FI cells at an E:T of 0.5:1. The number of donors tested is 3. NKTR-255 significantly enhanced the cytotoxic activity of MCAM CAR-engineered NK cells targeting NB SK-N-FI cells (A) and enhanced cytokine IFN-γ (B) and perforin (C) secretion from MCAM-CAR-NK cells. $^{*}p < 0.05$, $^{**}p < 0.01$. Columns represent mean values, and error bars indicate the SD of triplicate samples in a representative experiment. The same trend was seen in three independent biological replicates. (D) Schematic showing the *in vivo* study design and procedure in (E) and (F). NB SK-N-FI cells (4×10^6 cells/site) were implanted subcutaneously into 4- to 6-week-old female NSG mice. After tumor establishment, animals were divided into 4 groups and injected intraperitoneally with PBS or CAR NK cells in PBS (1×10^7/animal once a week for 5 weeks), NKTR-255 (0.3 mg/kg once every 2 weeks 3 times), or CAR NK cells combined with NKTR-255. Tumor growth was monitored by caliper measurement, and animals were followed until death or sacrificed upon reaching a tumor size of 2 cm in any dimension. (E) NKTR-255 further enhanced the efficacy of anti-MCAM-CAR-NK cells in significantly decreasing tumor growth in the NB xenograft mouse model. Tumor growth was monitored by caliper measurement twice a week. Tumor size was estimated according to the following formula: tumor size (cm^3) = length (cm) × width^2 (cm) × 0.5. $n = 7$ per group. $^{*}p < 0.05$, $^{**}p < 0.01$, $^{***}p < 0.001$ (ANOVA). Growth rates between groups were analyzed using a mixed-effects model. (F) Anti-MCAM-CAR-NK cell treatment combined with NKTR-255 further significantly prolonged animal survival compared to CAR NK cells alone in the NB xenograft mouse model. Mice were followed until death or sacrificed if any tumor size reached 2 cm in any dimension in (E). Probability of survival was determined by the Kaplan-Meier method using animal death/sacrifice as the terminal event using Prism v.8.0 (GraphPad). $^{*}p < 0.05$, $^{**}p < 0.01$, $^{***}p < 0.001$ (log rank test).

growth factor-reduced Matrigel matrix (Corning) were implanted subcutaneously into 4- to 6-week-old female NSG mice (The Jackson Laboratory). After tumor establishment, 1×10^7 of NK/CAR NK cells in PBS were injected intraperitoneally once a week for 5 weeks. We and others have shown that intraperitoneally injected NK cells are effective in solid tumor mouse models.[32] NKTR-255 (0.3 mg/kg) was injected intraperitoneally once every 2 weeks. To compare the anti-tumor efficacy of mock NK and CAR NK cells, NB xenograft tumor-bearing NSG mice were treated with mock NK cells, anti-MCAM-CAR-NK cells, or PBS control. To investigate the effect of CAR NK cells combined with NKTR-255 on NB xenograft tumor growth, tumor-bearing animals were treated with PBS control, CAR NK cells, NKTR-255, or CAR NK cells + NKTR-255. Before conducting mouse experiments, sample sizes achieving 80% power to detect an effect size >2 were determined at a significance level of 0.05. No randomization or blinding was used. Tumor growth was monitored by caliper measurement twice a week, as we have described previously.[28] Mice were followed until death or sacrificed upon reaching a tumor size of 2 cm in any dimension.

DATA AND CODE AVAILABILITY

Data and material are available upon reasonable request from mitchell_cairo@nymc.edu.

ACKNOWLEDGMENTS

The authors would like to thank Bin Liu, PhD (University of California, San Francisco, USA) for providing the scFv sequence; Carl June, MD, and Yangbing Zhao, MD, PhD (University of Pennsylvania, USA), for providing the CAR optimization construct; Nektar Therapeutics for providing NKTR-255; Nina Slivinsky, LVT, RLATG, for her technical assistance; and Ginny Davenport, RN, and Erin Morris, BSN, for their assistance with the preparation of this manuscript. This work was supported primarily by a grant from the National Cancer Institute Cancer Moonshot U54 CA232561 (to M.S.C., D.A.L., and T.P.C.). Additional support was from grants from the Pediatric Cancer Research Foundation and Children's Cancer Fund (to M.S.C.).

AUTHOR CONTRIBUTIONS

W.L., D.A.L., and M.S.C. conceived and designed the study. W.L., A.G., H.H., and X.M. developed the methodology, performed the analyses and interpreted the data. W.L., X.M., Y.C., T.P.C., and M.S.C. wrote, reviewed, and revised the manuscript. Y.C., M.T., Y.L., J.A., J.M.R., A.M.M., W.W.O., T.P.C., D.A.L., and M.S.C. provided administrative, technical and material support. All authors approved the final manuscript for submission.

DECLARATION OF INTERESTS

M.S.C. has served as a consultant for Jazz Pharmaceuticals, Omeros Pharmaceuticals, Servier Pharmaceuticals, Abbvie, and Novartis Pharmaceuticals; with the Speakers Bureau for Jazz Pharmaceuticals, Servier Pharmaceuticals, Amgen, Inc., Sanofi, and Sobi; and on the Advisory Board for Astra Zeneca and receives research funding from Celularity, Merck, Miltenyi Biotec, Servier, Omeros, Jazz, and Janssen. D.A.L. reports personal fees and others from Kiadis Pharma, CytoSen Therapeutics, Courier Therapeutics, and Caribou Biosciences outside of the submitted work. In addition, D.A.L. has a patent broadly related to NK cell therapy of cancer with royalties paid to Kiadis Pharma. T.P.C. recently served as a one-time consultant to Blueprint, Incyte, and Oncopeptides and as a DSMB chair for SpringWorks and is a cofounder of Vironexis Biotherapeutics, Inc.

SUPPLEMENTAL INFORMATION

Supplemental information can be found online at https://doi.org/10.1016/j.omton.2024.200894.

REFERENCES

1. Yan, P., Qi, F., Bian, L., Xu, Y., Zhou, J., Hu, J., Ren, L., Li, M., and Tang, W. (2020). Comparison of Incidence and Outcomes of Neuroblastoma in Children, Adolescents, and Adults in the United States: A Surveillance, Epidemiology, and End Results (SEER) Program Population Study. Med. Sci. Monit. *26*, e927218.

2. Whittle, S.B., Smith, V., Doherty, E., Zhao, S., McCarty, S., and Zage, P.E. (2017). Overview and recent advances in the treatment of neuroblastoma. Expert Rev. Anticancer Ther. *17*, 369–386.

3. Maris, J.M. (2010). Recent advances in neuroblastoma. N. Engl. J. Med. *362*, 2202–2211.

4. Ozkaynak, M.F., Gilman, A.L., London, W.B., Naranjo, A., Diccianni, M.B., Tenney, S.C., Smith, M., Messer, K.S., Seeger, R., Reynolds, C.P., et al. (2018). A Comprehensive Safety Trial of Chimeric Antibody 14.18 With GM-CSF, IL-2, and Isotretinoin in High-Risk Neuroblastoma Patients Following Myeloablative Therapy: Children's Oncology Group Study ANBL0931. Front. Immunol. *9*, 1355.

5. Park, J.R., Kreissman, S.G., London, W.B., Naranjo, A., Cohn, S.L., Hogarty, M.D., Tenney, S.C., Haas-Kogan, D., Shaw, P.J., Kraveka, J.M., et al. (2019). Effect of Tandem Autologous Stem Cell Transplant vs Single Transplant on Event-Free Survival in Patients With High-Risk Neuroblastoma: A Randomized Clinical Trial. JAMA *322*, 746–755.

6. Yu, A.L., Gilman, A.L., Ozkaynak, M.F., London, W.B., Kreissman, S.G., Chen, H.X., Smith, M., Anderson, B., Villablanca, J.G., Matthay, K.K., et al. (2010). Anti-GD2 antibody with GM-CSF, interleukin-2, and isotretinoin for neuroblastoma. N. Engl. J. Med. *363*, 1324–1334.

7. Cho, D., Shook, D.R., Shimasaki, N., Chang, Y.H., Fujisaki, H., and Campana, D. (2010). Cytotoxicity of activated natural killer cells against pediatric solid tumors. Clin. Cancer Res. *16*, 3901–3909.

8. Chu, Y., Hochberg, J., Yahr, A., Ayello, J., van de Ven, C., Barth, M., Czuczman, M., and Cairo, M.S. (2015). Targeting CD20+ Aggressive B-cell Non-Hodgkin Lymphoma by Anti-CD20 CAR mRNA-Modified Expanded Natural Killer Cells In Vitro and in NSG Mice. Cancer Immunol. Res. *3*, 333–344.

9. Liu, E., Marin, D., Banerjee, P., Macapinlac, H.A., Thompson, P., Basar, R., Nassif Kerbauy, L., Overman, B., Thall, P., Kaplan, M., et al. (2020). Use of CAR-Transduced Natural Killer Cells in CD19-Positive Lymphoid Tumors. N. Engl. J. Med. *382*, 545–553.

10. Orentas, R.J., Yang, J.J., Wen, X., Wei, J.S., Mackall, C.L., and Khan, J. (2012). Identification of cell surface proteins as potential immunotherapy targets in 12 pediatric cancers. Front. Oncol. *2*, 194.

11. Esser, R., Müller, T., Stefes, D., Kloess, S., Seidel, D., Gillies, S.D., Aperlo-Iffland, C., Huston, J.S., Uherek, C., Schönfeld, K., et al. (2012). NK cells engineered to express a GD2 -specific antigen receptor display built-in ADCC-like activity against tumour cells of neuroectodermal origin. J. Cell Mol. Med. *16*, 569–581.

12. Sechler, M., Parrish, J.K., Birks, D.K., and Jedlicka, P. (2017). The histone demethylase KDM3A, and its downstream target MCAM, promote Ewing Sarcoma cell migration and metastasis. Oncogene *36*, 4150–4160.

13. Wang, J., Tang, X., Weng, W., Qiao, Y., Lin, J., Liu, W., Liu, R., Ma, L., Yu, W., Yu, Y., et al. (2015). The membrane protein melanoma cell adhesion molecule (MCAM) is a novel tumor marker that stimulates tumorigenesis in hepatocellular carcinoma. Oncogene *34*, 5781–5795.

14. McGary, E.C., Heimberger, A., Mills, L., Weber, K., Thomas, G.W., Shtivelband, M., Lev, D.C., and Bar-Eli, M. (2003). A fully human antimelanoma cellular adhesion molecule/MUC18 antibody inhibits spontaneous pulmonary metastasis of osteosarcoma cells *in vivo*. Clin. Cancer Res. *9*, 6560–6566.

15. Miyazaki, T., Maiti, M., Hennessy, M., Chang, T., Kuo, P., Addepalli, M., Obalapur, P., Sheibani, S., Wilczek, J., Pena, R., et al. (2021). NKTR-255, a novel polymer-conjugated rhIL-15 with potent antitumor efficacy. J. Immunother. Cancer *9*, e002024.

16. Shah, N., Perales, M.A., Turtle, C.J., Cairo, M.S., Cowan, A.J., Saeed, H., Budde, L.E., Tan, A., Lee, Z., Kai, K., et al. (2021). Phase I study protocol: NKTR-255 as monotherapy or combined with daratumumab or rituximab in hematologic malignancies. Future Oncol. *17*, 3549–3560.

17. Richards, R.M., Sotillo, E., and Majzner, R.G. (2018). CAR T Cell Therapy for Neuroblastoma. Front. Immunol. *9*, 2380.

18. Richman, S.A., Nunez-Cruz, S., Moghimi, B., Li, L.Z., Gershenson, Z.T., Mourelatos, Z., Barrett, D.M., Grupp, S.A., and Milone, M.C. (2018). High-Affinity GD2-Specific CAR T Cells Induce Fatal Encephalitis in a Preclinical Neuroblastoma Model. Cancer Immunol. Res. *6*, 36–46.

19. Sivori, S., Parolini, S., Marcenaro, E., Castriconi, R., Pende, D., Millo, R., and Moretta, A. (2000). Involvement of natural cytotoxicity receptors in human natural killer cell-mediated lysis of neuroblastoma and glioblastoma cell lines. J. Neuroimmunol. *107*, 220–225.

20. Melaiu, O., Chierici, M., Lucarini, V., Jurman, G., Conti, L.A., De Vito, R., Boldrini, R., Cifaldi, L., Castellano, A., Furlanello, C., et al. (2020). Cellular and gene signatures of tumor-infiltrating dendritic cells and natural-killer cells predict prognosis of neuroblastoma. Nat. Commun. *11*, 5992.

21. Nayyar, G., Chu, Y., and Cairo, M.S. (2019). Overcoming Resistance to Natural Killer Cell Based Immunotherapies for Solid Tumors. Front. Oncol. *9*, 51.

22. Denman, C.J., Senyukov, V.V., Somanchi, S.S., Phatarpekar, P.V., Kopp, L.M., Johnson, J.L., Singh, H., Hurton, L., Maiti, S.N., Huls, M.H., et al. (2012). Membrane-bound IL-21 promotes sustained ex vivo proliferation of human natural killer cells. PLoS One *7*, e30264.

23. Chu, Y., Yahr, A., Huang, B., Ayello, J., Barth, M., and S Cairo, M. (2017). Romidepsin alone or in combination with anti-CD20 chimeric antigen receptor expanded natural killer cells targeting Burkitt lymphoma *in vitro* and in immunodeficient mice. OncoImmunology *6*, e1341031.

24. Foster, J.B., Griffin, C., Rokita, J.L., Stern, A., Brimley, C., Rathi, K., Lane, M.V., Buongervino, S.N., Smith, T., Madsen, P.J., et al. (2022). Development of

GPC2-directed chimeric antigen receptors using mRNA for pediatric brain tumors. J. Immunother. Cancer *10*, e004450.

25. He, W.G., Yan, Y., Tang, W., Cai, R., and Ren, G. (2017). Clinical and biological features of neuroblastic tumors: A comparison of neuroblastoma and ganglioneuroblastoma. Oncotarget *8*, 37730–37739.

26. Fry, T.J., Shah, N.N., Orentas, R.J., Stetler-Stevenson, M., Yuan, C.M., Ramakrishna, S., Wolters, P., Martin, S., Delbrook, C., Yates, B., et al. (2018). CD22-targeted CAR T cells induce remission in B-ALL that is naive or resistant to CD19-targeted CAR immunotherapy. Nat. Med. *24*, 20–28.

27. Tang, F., Zhao, L.T., Jiang, Y., Ba, D.N., Cui, L.X., and He, W. (2008). Activity of recombinant human interleukin-15 against tumor recurrence and metastasis in mice. Cell. Mol. Immunol. *5*, 189–196.

28. Chu, Y., Nayyar, G., Jiang, S., Rosenblum, J.M., Soon-Shiong, P., Safrit, J.T., Lee, D.A., and Cairo, M.S. (2021). Combinatorial immunotherapy of N-803 (IL-15 superagonist) and dinutuximab with ex vivo expanded natural killer cells significantly enhances *in vitro* cytotoxicity against GD2(+) pediatric solid tumors and *in vivo* survival of xenografted immunodeficient NSG mice. J. Immunother. Cancer *9*, e002267.

29. Bidlingmaier, S., He, J., Wang, Y., An, F., Feng, J., Barbone, D., Gao, D., Franc, B., Broaddus, V.C., and Liu, B. (2009). Identification of MCAM/CD146 as the target antigen of a human monoclonal antibody that recognizes both epithelioid and sarcomatoid types of mesothelioma. Cancer Res. *69*, 1570–1577.

30. Zhao, Y., Moon, E., Carpenito, C., Paulos, C.M., Liu, X., Brennan, A.L., Chew, A., Carroll, R.G., Scholler, J., Levine, B.L., et al. (2010). Multiple injections of electroporated autologous T cells expressing a chimeric antigen receptor mediate regression of human disseminated tumor. Cancer Res. *70*, 9053–9061.

31. Sanjana, N.E., Shalem, O., and Zhang, F. (2014). Improved vectors and genome-wide libraries for CRISPR screening. Nat. Methods *11*, 783–784.

32. Geller, M.A., Knorr, D.A., Hermanson, D.A., Pribyl, L., Bendzick, L., McCullar, V., Miller, J.S., and Kaufman, D.S. (2013). Intraperitoneal delivery of human natural killer cells for treatment of ovarian cancer in a mouse xenograft model. Cytotherapy *15*, 1297–1306.

OMTON, Volume *32*

Supplemental information

Combinatorial immunotherapy of anti-MCAM CAR-modified expanded natural killer cells and NKTR-255 against neuroblastoma

Wen Luo, Aliza Gardenswartz, Hai Hoang, Yaya Chu, Meijuan Tian, Yanling Liao, Janet Ayello, Jeremy M. Rosenblum, Xiaokui Mo, A. Mario Marcondes, Willem W. Overwijk, Timothy P. Cripe, Dean A. Lee, and Mitchell S. Cairo

Supplemental Information

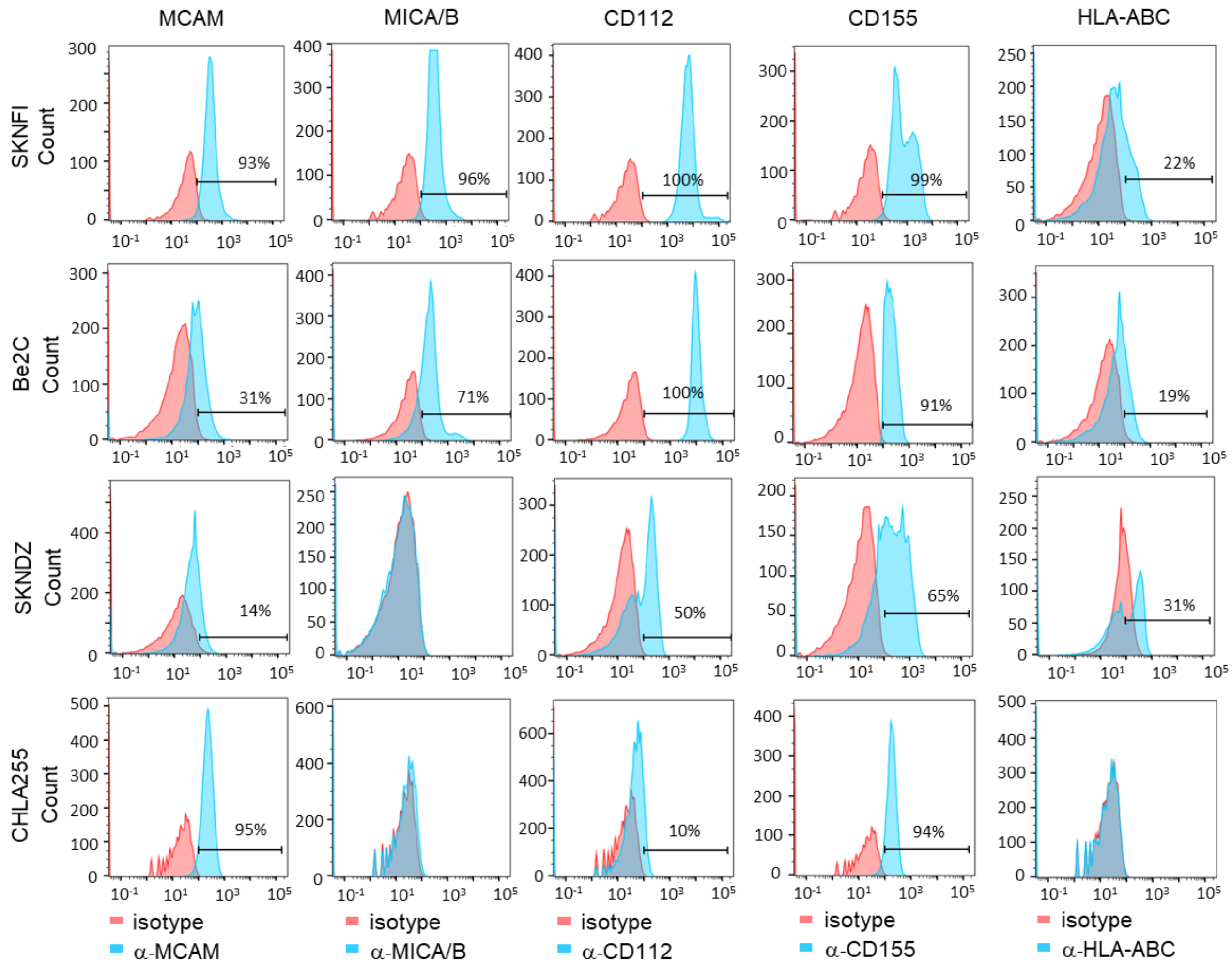

Figure S1. Representative flow histograms of MCAM and NK receptor ligands showing expression on the surface of NB cell lines. SK-N-FI, Be2C, SK-N-DZ and CHLA255 cells were stained with isotypes or flow antibodies against MCAM or NK activating receptor ligands MICA/B, CD112, CD155, or NK inhibitory receptor ligands HLA-ABC, and subject to flow cytometry analyses.

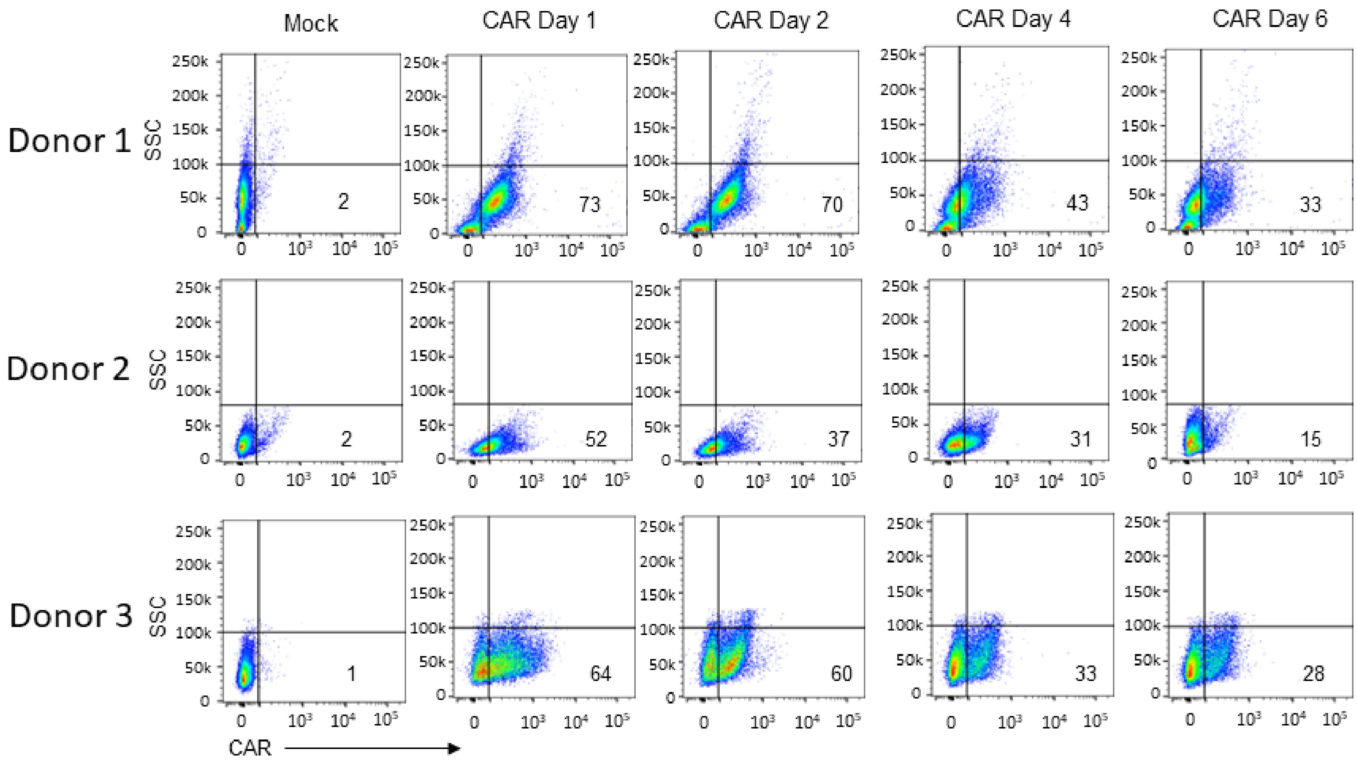

Figure S2. Electroporation mediated CAR expression on ex-vivo expanded NK cells from 3 different donors. Ten micrograms of in vitro transcribed CAR mRNA were introduced into 5×10^6 exNK cells using electroporation. Twenty four to 48 hours after electroporation, CAR expression was detected by biotinylated MCAM protein followed by FITC-streptavidin staining and flow cytometry. SSC-FITC dot plots are shown. The numbers are percent of CAR positive population in the total cells.

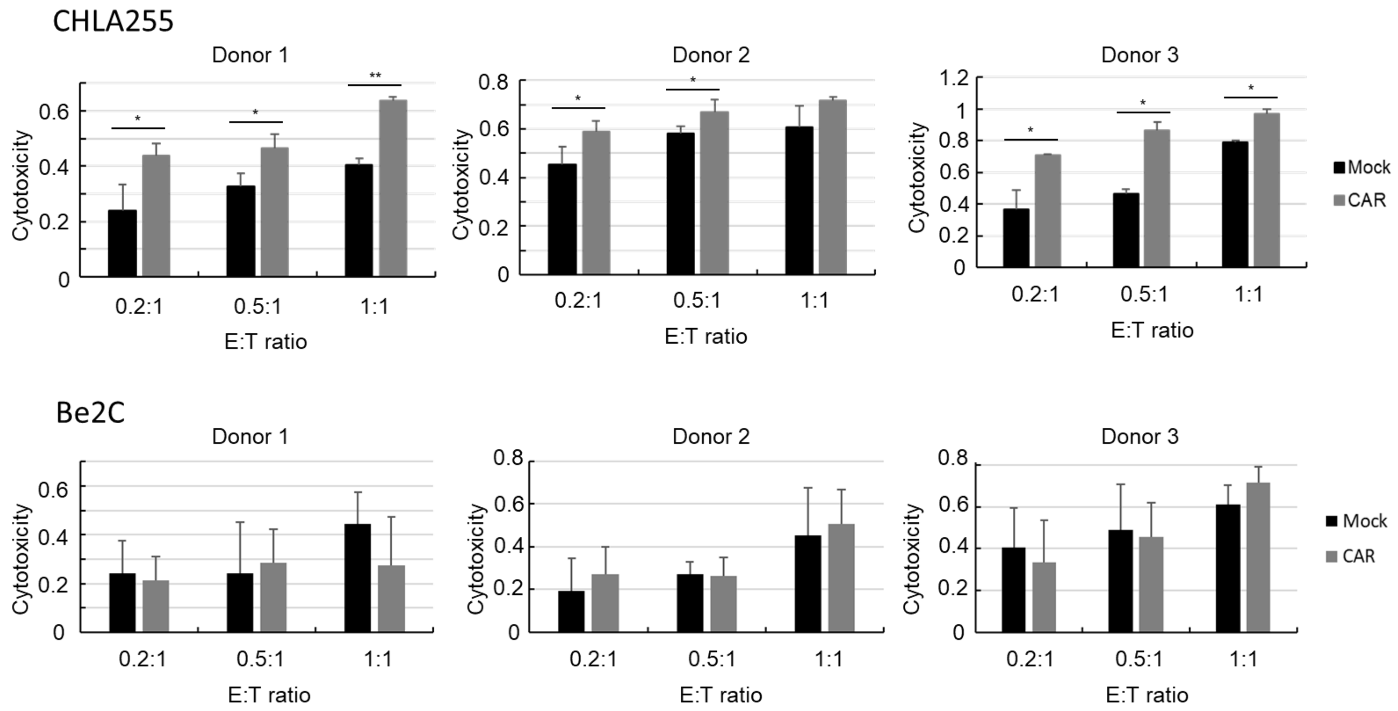

Figure S3. MCAM CAR NK cell cytotoxic activity against NB CHLA255 and Be2C cells compared to the mock NK cells. Columns represent the mean values; error bars indicate the standard deviation of triplicate samples in a representative experiment. *$p < 0.05$, **$p < 0.01$. The same trend was seen using three different donors derived NK cells.

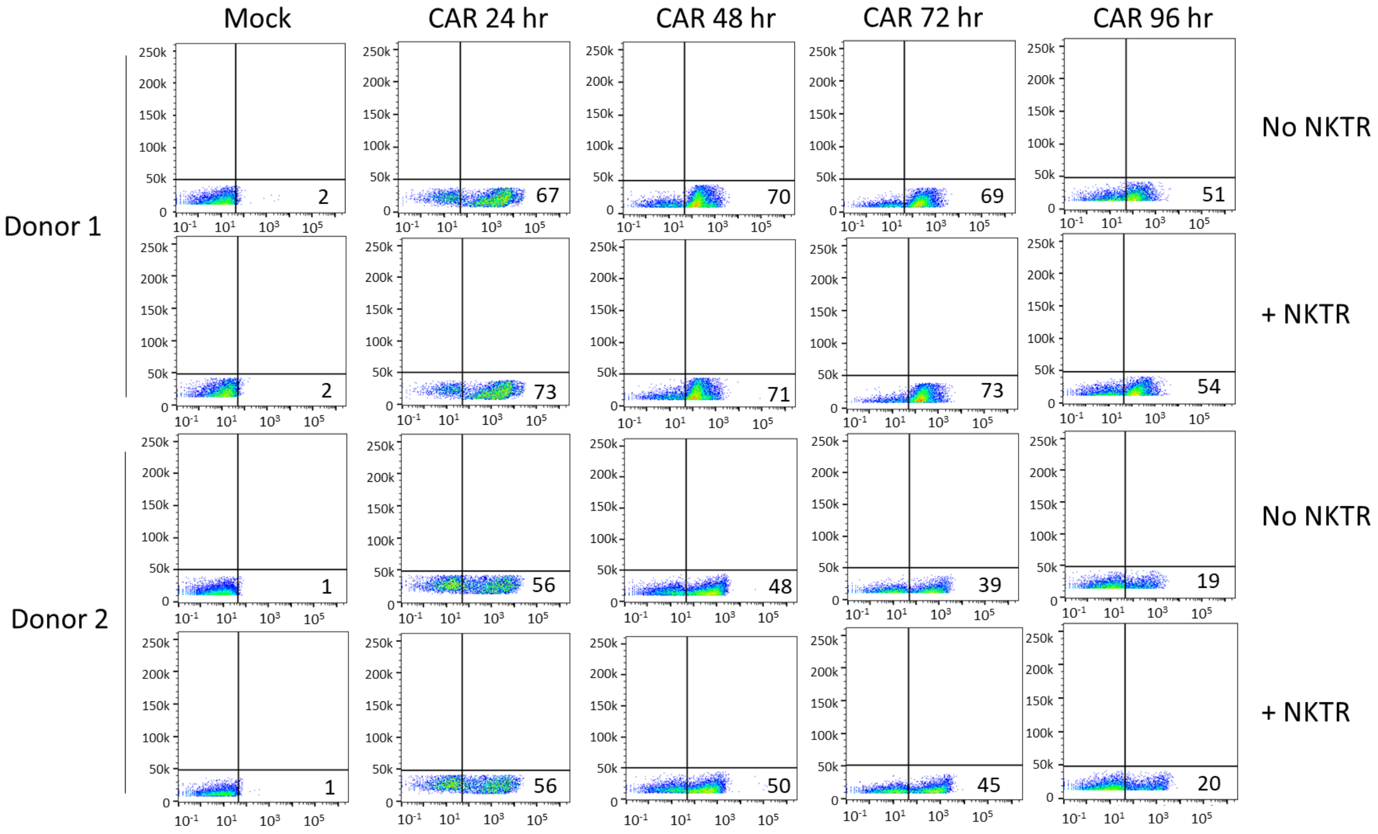

Figure S4. Effects of NKTR-255 on MCAM CAR expression level and duration on ex-vivo expanded NK cells. NK cells were electroporated with CAR mRNA and incubated with NKTR-255 (40 ng/mL) in the absence of IL-2 in RPMI media for 96 hours. CAR NK cells were sampled every 24 hours and CAR expression was detected by biotinylated MCAM protein followed by FITC-streptavidin staining and flow cytometry. SSC-FITC dot plots are shown. The numbers are the percent of CAR positive population in the total cells.

Supplemental Material

Cell lines

NB cell lines SK-N-FI, Be2C and SK-N-DZ were obtained from American Type Culture Collection (ATCC) and grown according to ATCC recommendations. CHLA-255 cells were

generously provided by Robert Seeger, MD from Children's Hospital Los Angeles, CA, USA. Cell lines are tested quarterly for mycoplasma contamination using MycoProbe Mycoplasma Detection Kit (CUL001B, R&D Systems, Minneapolis, MN, USA) and cells are authenticated by short tandem repeat profiling (Genetica DNA Laboratories, Cincinnati, OH, USA).

DNA constructs

The MCAM Ab single chain variable fragment (scFv) sequence was generously provided by Bin Liu (University of California at San Francisco, United States)[29] and the scFv DNA was codon optimized and synthesized (Integrated DNA Technologies, Coralville, IA, USA) followed by subcloning in-frame with a linker (eskygppcppcpm), CD28TM (NP_001230007.1, aa 34-59), 4-1BB (NP_001552, aa 213-255), and CD3ζ (NP_000725, aa 52-163) into the pcDNA3 vector to generate a second generation anti-MCAM CAR. A 2bgUTR.150A sequence generously provided by Carl June and Yangbing Zhao (University of Pennsylvania)[30] was inserted at 3' end of the CAR construct to further optimize the construct. The clustered regulatory interspaced short palindromic repeats (CRISPR)/Cas9 knockout constructs for MCAM were created by cloning the CRISPR guide RNA against MCAM (5'-GTTGCATGACCTGAAACGGG-3' and 5'-AGGAGGCGGCTATCGCTGCG-3') into the plentiCRISPRv2 vector (Addgene plasmid #52961).[31] Guide sequences were designed using the Broad Institute sgRNA designer tool (https://portals.broadinstitute.org/gpp/public/analysis-tools/sgrna-design).

Ex-vivo NK expansion

NK cells were expanded using donor peripheral blood mononuclear cells and irradiated K562-mbIL21-41BBL feeder cells and purified by using a NK cell isolation kit (Miltenyi Biotec, 130-092-657, Bergisch Gladbach, Germany) as we have previously described.[8]

CAR mRNA electroporation

Anti-MCAM CAR mRNA was in-vitro transcribed using the mMESSAGE mMACHINE T7 ultra transcription kit (Thermo Fisher Scientific, AM1345, Waltham, MA, USA) according to the manufacturer's instructions. Expanded NK cells ($5x10^6$) were electroporated with anti-MCAM CAR mRNA (10 µg) or H_2O (negative control) using the MaxCyte GT® electroporation system (MaxCyte Inc. Rockville, MD, USA). Anti-MCAM CAR mRNA electroporation efficiency was evaluated by flow cytometry using biotinylated MCAM protein (HY-P75613, MedChemExpress, Monmouth Junction, NJ, USA) followed by FITC-streptavidin (405202, BioLegend, San Diego, CA, USA). Anti-MCAM CAR expression was detected at day 1, 2, 4, and 6 post electroporation.

Bioluminescence (BLI) based in-vitro cytotoxicity assay

BLI based in-vitro cytotoxicity assays were performed as we have previously described[27] with minor modifications. Luciferase-expressing tumor cells ($5x10^4$) were incubated with effector cells (NK/CAR NK) at different effector-to-target (E:T) ratios (0.2:1, 0.5:1, and 1:1) in DMEM media supplemented with 10% FBS in 96–well tissue culture plates at 37°C for 4 hours before D-firefly luciferin potassium salt (LUCK-1G, Goldbio, St Louis, MO, USA) was added to the cells and BLI was measured with a luminometer (Molecular Devices Multifilter F5 plate reader). In CAR NK and NKTR-255 combination cytotoxicity assay, MCAM CAR NK cells were cultured in RPMI1640 media supplemented with or without NKTR-255 (40 ng/ml, Nektar

Therapeutics, San Francisco, CA, USA) for 72 hours before incubating with luciferase expressing tumor cells.

Flow cytometry

Cells (1x10^6 single cell suspension) were washed once with ice cold FACS buffer (DPBS, 0.5% BSA) and blocked with 100 µL of Fc block (BD Biosciences, 564219, Franklin Lakes, NJ, USA) diluted in FACS buffer at 1:50 ratio at 4°C for 20 min. Cells were then collected by centrifugation at 1500 rpm for 5 min and resuspended in 100 µL fluorescent conjugated primary antibody in FACS buffer (for antibody concentration see Supplementary Table 1) and incubated at 4°C for 1 hour in the dark. Cells were washed twice and resuspended in 100 µL ice cold FACS buffer. If cells were stained with unlabeled primary antibody, cells were resuspended in fluorescent conjugated secondary antibody in FACS buffer at the dilution ratio recommended by the manufacturer (see Supplementary Table 1 for details) and incubated at 4°C in the dark for 30 min. Cells were then washed twice and resuspended in 100 µL ice cold FACS buffer. Flow cytometry analyses were performed using FACSCelesta Cell Analyzer and data were analyzed by FACSDeva (BD Biosciences, Franklin Lakes, NJ, USA) and Flowjo (FlowJo LLC, Ashland, OR, USA).

Enzyme-linked immunosorbent assay (ELISA)

IFN-γ (430104, BioLegend, San Diego, CA, USA) and Perforin (ab46068, Abcam, Cambridge, UK) concentrations were analyzed by ELISA according to the manufacturer's instructions as we have previously described.[27]

In-vitro treatment of NK cells with NKTR-255

ExNK cells were isolated from day 14 of expansion culture (using IL-2 and irradiated feeder cells) from three donors by NK cell isolation kit (Miltenyi Biotec, 130-092-657, Bergisch Gladbach, Germany). Isolated NK cells ($2.5x10^6$ per condition) were then incubated with 0, 1, 10, or 40 ng/mL of NKTR-255 in RPMI 1640 media for 6 days. The number of viable cells were counted every 24 hours using trypan blue staining method.

Statistical analyses

Analysis of variance (ANOVA) was used to analyze experiments with multiple independent groups. In-vivo tumor growth was analyzed by mixed effect model, accounting for observational dependencies for each subject. ANOVA and mixed effect modeling were conducted using SAS 9.4 (SAS Institute, Cary, NC, USA). Before conducting mouse experiments, sample sizes achieving 80% power to detect an effect size >2 were determined at significant level as 0.05 using PASS 20 (Power Analysis and Sample Size Software. NCSS, LLC.). All data are presented as the mean ± SD of at least three independent experiments except where stated.
